# Readability of Online Patient Education Materials for Myopia Management

**DOI:** 10.1007/s44402-026-00030-6

**Published:** 2026-03-16

**Authors:** Monica Jong, Michelle Waugh, Pinar Ozmizrak, Ian Flitcroft

**Affiliations:** 1 Optometry and Vision Science, University of New South Wales Medicine & Health, Sydney, New South Wales Australia; 2 Freelance Medical Writer, Melbourne, Australia; 3 Freelance Medical Writer, Ottawa, Canada; 4 Technological University Dublin, Dublin, Ireland; 5 Children’s University Hospital, University College Dublin, Dublin, Ireland

**Keywords:** Myopia, Myopia management, Online health information, Patient education, Readability, Refractive error

## Abstract

**Purpose:**

Patients are turning to the internet to access educational materials to help them make healthcare decisions, making readability an important factor. This cross-sectional study assessed the readability of online patient education materials for myopia management treatments that have regulatory approval.

**Methods:**

The top 10 Google search results from May 2024 for freely available online patient information on myopia management modalities and regulatory-approved products in Canada and Australia were analysed for readability. The modalities included orthokeratology, myopia control spectacle lenses, myopia control soft contact lenses and atropine. The products included MiYOSMART® [HOYA®], Stellest® [Essilor®], MyoCare® [ZEISS], MiSight® 1 day [CooperVision®], ACUVUE® Abiliti® 1-Day [Johnson & Johnson], NaturalVue® Multifocal 1 Day [VTI], ACUVUE® Abiliti® Overnight [Johnson & Johnson] and Eikance [Aspen Pharmacare Australia]. These searches gave 120 results. Readability was assessed with Flesch Reading Ease Score (FRES), Flesch-Kincaid Grade Level (FKGL), Gunning Fog Index (GFI), Simple Measure of Gobbledygook (SMOG) Index and Coleman Liau Index (CLI). Additionally, websites were scored on *Journal of the American Medical Association* (*JAMA*) benchmark criteria. Statistical analysis was performed with two-tailed tests.

**Results:**

Of 120 websites, none met the recommended sixth-grade reading level across all readability indices. There were 13 websites meeting at least one readability index, 10 being product-related. There were seven websites satisfying all four *JAMA* benchmarks, while the majority met one. There was a weak positive relationship between product search rank and readability (SMOG *p* = 0.02, GFI *p* = 0.02) and a weak negative relationship between *JAMA* benchmarks and readability for both modality (CLI *p* = 0.045) and product (CLI *p* = 0.049).

**Conclusions:**

Online information about myopia management is generally written above the recommended sixth-grade reading level and does not meet all *JAMA* benchmarks. Websites that appear as top search results do not necessarily have easier readability. The readability of online patient education materials may influence access to treatment and outcomes.

Key Points

- Online patient education materials on myopia management are often written above the recommended sixth-grade reading level.
- Top-ranking search results of myopia management websites are not necessarily more readable.
- Improving readability can support information accessibility and awareness of myopia management.

## Introduction

Myopia is estimated to affect 50% of the world’s population by 2050 and has been recognised by the World Health Organization as a public health issue [1]. The World Society of Paediatric Ophthalmology and Strabismus and American Academy of Ophthalmology (AAO) have also acknowledged that myopia management should be the standard of care [2, 3]. Patients are increasingly seeking education about their health and how to manage it [4]. Approximately 69–81.5% of US adults reported using the internet first during their most recent search for health information in studies conducted from 2008 to 2018 [5], and patients and caregivers may use this information to inform their medical decisions [6]. A study found a similar proportion of Europeans (79%) used the internet to seek health information [7]. Therefore, it is both useful and pertinent for eye care practitioners to understand patient education materials and to be prepared to review the information with patients. Research has shown that involving patients in clinical decisions (i.e., shared decision-making), offering patient education and understanding the problems and needs of patients are important aspects that contribute to the success of treatment adherence [8–10].

There is a global burden from lost productivity directly and indirectly caused by uncorrected myopia, as well as potential permanent vision loss due to ocular diseases associated with increasing myopia [11–13]. Despite current prescribing trends showing that eye care practitioners are prescribing more management treatments [14] to mitigate the myopia epidemic [1], the general awareness of myopia among parents remains low [15, 16]. A survey that assessed parental attitudes to myopia found that most parents were not aware of the health risks associated with myopia [15]. Eye care practitioners should play a pivotal role in communicating to parents and caregivers of children with myopia the need to manage myopia, as well as offering and implementing myopia management options [10].

Myopia management treatments are offered based on a child’s myopia risk profile, lifestyle factors and perceived ability to tolerate or handle a treatment [17]. Additionally, four key factors, i.e., compliance, quality of vision, quality of life and safety, are some of the fundamental considerations when comparing overall efficacy and prescribing a myopia management plan [18].

Further, it has been found that information retrieved on the internet is critical to patients’ decision-making [19, 20], and those who obtain such health information are more able to follow the treatment process and experience better therapeutic treatment effects [21]. The usefulness of health information depends, in part, upon the readability of patient education materials. The US Department of Health and Human Services (USDHHS) recommends patient education materials be written at or below a sixth-grade (corresponding to 11–12 years of age) reading level. Additionally, the Agency for Healthcare Research and Quality, a US government agency, recommends that written materials aiming to improve personal health literacy should be at a fourth- to sixth-grade (corresponding to 9–12 years of age) level [22].

The objective of this cross-sectional study was to analyse the readability of online patient education materials related to approved myopia management treatments using standardised and validated metrics.

## Methods

In order to assess the readability of online patient education materials for myopia management modalities and products, the top 10 Google (google.com) search results for each modality were analysed. These modalities included orthokeratology, myopia control spectacle lenses, myopia control soft contact lenses, atropine and repeated low-level red-light therapy. The products included MiYOSMART® [HOYA®, hoyavision.com] (spectacles), Stellest® [Essilor®, essilorluxottica.com] (spectacles), MyoCare® [ZEISS, zeiss.com] (spectacles), MiSight® 1 day [CooperVision®, coopervision.com] (soft contact lenses), ACUVUE® Abiliti® 1-Day [Johnson & Johnson, jnj.com] (soft contact lenses), NaturalVue® Multifocal 1 Day [VTI, vtivision.com] (soft contact lenses), ACUVUE® Abiliti® Overnight [Johnson & Johnson, jnj.com] (orthokeratology), Eikance [Aspen Pharmacare Australia, aspenpharma.com.au] (atropine) and Eyerising [Eyerising International, eyerisinginternational.com] (repeated low-level red-light therapy). The searches were performed in May 2024.

### Search Methodology

Google searches were conducted in two separate regions: Canada and Australia. These regions were chosen as they have a high proportion of myopia management treatments available and require regulatory approval. Search regions were specified because the origin of a search can affect the order of Google search results, but results were not limited to websites from Canada or Australia. Selections were restricted to the first 20 listed websites on the Google search engine, which approximately equates to the first two pages of Google search results. From these 20 websites, the first 10 to meet the inclusion criteria were collected.

In order to yield the most relevant modality-related webpages first, a narrow Google search was conducted with the keywords ‘myopia control’ OR ‘myopia management’ AND + ‘*modality type*’ (e.g., orthokeratology) AND ‘patient information’. To broaden the search, the second search consisted of the keywords ‘myopia control’ OR ‘myopia management’ AND + ‘*modality type’* (e.g., orthokeratology) and also a separate search with the ‘*modality type*’ and ‘patient information’ together.

Searches for the modalities orthokeratology and soft contact lenses were conducted within Canada. Searches for the modalities atropine and spectacles lenses were conducted within Australia, as the Australian search region was able to yield a minimum of 10 patient-facing online website results threshold. To yield the most relevant product-related webpages first, a narrow Google search was conducted with the keywords ‘myopia control’ OR ‘myopia management’ AND + ‘*product name*’ (e.g., MiYOSMART) AND ‘patient information’. Afterwards, the search was broadened by using only the product name.

Since Canada has the highest number of regulated products, all product-specific searches were conducted within the Canadian search region, except for Eikance (an atropine medication) and Eyerising (a repeated low-level red-light device), which have regulatory approval in Australia; hence, these searches were conducted within Australia.

### Inclusion and Exclusion Criteria

To be included in the analysis, websites had to be in English and be patient-oriented resources. Websites could be based in any country. PDFs found on the websites (i.e., patient brochures, product patient information guides, product package inserts or patient information guides) were also included. Journal publications and websites directed at practitioners were excluded.

### Readability Analysis

Readability analysis was conducted in Readable, an online text-scoring tool [23], using the Flesch Reading Ease Score (FRES) [24], Flesch-Kincaid Grade Level (FKGL) [25], Gunning Fog Index (GFI) [26], Simple Measure of Gobbledygook (SMOG) Index [27] and Coleman Liau Index (CLI) [28]. These formulas have been used in several previous studies assessing the readability of online patient education materials [29–31]. The FRES formula assigns a score to the text from 0 to 100 by factoring in total words, sentences and syllables. The higher the score, the easier it is to read (e.g., a score of 80–90 is equivalent to a sixth-grade reading level). The FKGL formula considers the same factors as the FRES formula, but weights those factors differently. The FKGL, GFI, SMOG and CLI provide a grade level between 0 and 20; thus, a text with a score of 6 should be readable by those educated to sixth grade in the US (6 years of formal schooling, corresponding to 11–12 years of age). The GFI score emphasises complex, polysyllabic words as well as the total number of sentences. Alternatively, the SMOG score counts the number of polysyllabic words in a fixed number of sentences. The CLI score examines the average number of letters per 100 words and the average number of sentences per 100 words. The USDHHS sixth-grade reading level recommendation corresponds with a FRES score ≥80, and FKGL, GFI, SMOG and CLI scores ≤6.9.

### *JAMA* Benchmark Analysis

The *Journal of the American Medical Association* (*JAMA*) criteria comprise four benchmarks: attribution, authorship, currency and disclosure [30]. To comply with these benchmark requirements, websites had to include the following information: references and copyright information for attribution; contributing authors, their credentials and affiliations for authorship; date posted and last updated for currency (recency of information) and website ownership, sponsorship, advertising and funding for disclosure.

### Statistical Analysis

Statistical analysis was performed with IBM SPSS Statistics Version 29.0.0.0 (ibm.com). Summary statistics were used to describe the readability scores for modalities and products. Kruskal–Wallis tests with Dunn’s post-hoc analysis adjusted by the Bonferroni correction for multiple tests (*p* < 0.05) were employed to compare readability scores between modalities and between products. Pearson correlation tests (*p* < 0.05) were utilised to evaluate correlations between the number of *JAMA* benchmarks and readability score, as well as between the search result rank and readability score.

## Results

### Website Selection

A total of 120 freely available online resources were found, with 40 being myopia management modality search results (Appendix Table 1) and 80 being myopia management product search results (Appendix Table 2). The results for the repeated low-level red-light therapy modality and Eyerising product were eliminated from this analysis as 10 patient education websites were unavailable on Google at the time of the search.

### Myopia Management Modalities

The median scores for the 40 treatment modality queries were as follows: FRES 51.2 (interquartile range (IQR): 7.9, range 35.1–66.5); FKGL 9.9 (IQR: 2.8, range 6.3–13.0); GFI 12.1 (IQR: 3.6, range 7.8–15.7); SMOG 12.5 (IQR: 2.5, range 8.9–14.9); and CLI 12.0 (IQR: 2.1, range 8.5–13.6) (Table 1).

**Table 1:** Readability scores of patient education materials related to myopia modalities.

	Median readability scores (IQR) [range]						
Modality	Word count	Words/Sentence	FRES	FKGL	GFI	SMOG	CLI
Orthokeratology	1319 (1021)	13.9 (6.0)	49.6 (14.8)	9.8 (3.1)	11.3 (3.3)	11.8 (2.6)	11.1 (2.7)
	[622–8167]	[5.3–18.7]	[35.1–62.9]	[7.1–13.0]	[8.1–15.7]	[8.9–14.9]	[8.7–13.6]
Spectacles	1042 (975)	13.4 (5.1)	52.2 (7.4)	9.9 (2.5)	11.9 (2.9)	12.6 (1.9)	12.0 (1.5)
	[284–2345]	[6.6–16.5]	[40.6–62.9]	[6.8–10.6]	[8.6–13.4]	[9.8–13.2]	[9.6–13.4]
Atropine	1051 (563)	14.6 (3.8)	50.8 (4.0)	9.8 (1.5)	12.5 (1.5)	12.8 (0.9)	11.9 (1.1)
	[654–2566]	[10.1–18.3]	[42.5–63.1]	[7.0–11.8]	[8.1–14.9]	[10.0–14.9]	[9.4–12.9]
Soft contact lenses	778 (660)	15.0 (4.3)	50.6 (12.2)	10.0 (2.4)	12.1 (2.8)	12.4 (2.1)	12.2 (1.4)
	[341–2036]	[6.0–19.1]	[43.0–66.5]	[6.3–11.8]	[7.8–14.0]	[9.2–14.2]	[8.5–13.5]
*All modalities*	1042 (889)	14.5 (4.6)	51.2 (7.9)	9.9 (2.8)	12.1 (3.6)	12.5 (2.5)	12.0 (2.1)
	[284–8167]	[5.3–19.1]	[35.1–66.5]	[6.3–13.0]	[7.8–15.7]	[8.9–14.9]	[8.5–13.6]

*FKGL* Flesch-Kincaid Grade Level, *FRES* Flesch Reading Ease Score, *GFI* Gunning Fog Index, *SMOG* Simple Measure of Gobbledygook Index, *CLI* Coleman Liau Index, *IQR* interquartile range.

**Fig. 1:**
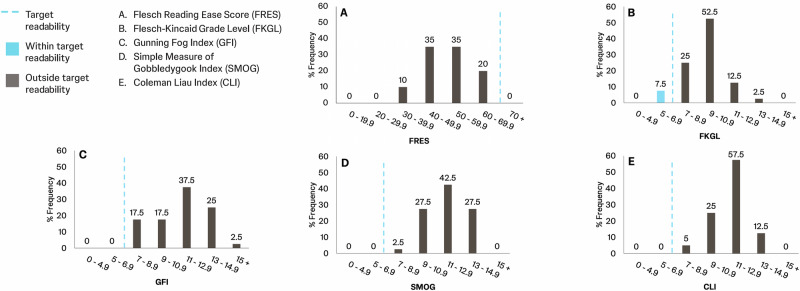
Percent frequency distribution of readability scores of online patient education materials for myopia management modalities. Blue bars indicate meets target sixth-grade readability level; grey bars indicate does not meet sixth-grade readability.

Of all the myopia modality-related search results, 7.5% (3/40) were found to be compliant with the FKGL score, with no sites meeting the FRES, GFI, SMOG, or CLI score targets (i.e., the sixth-grade reading level recommendation made by the USDHHS; corresponding with a FRES score ≥80 and FKGL, SMOG, GFI and CLI scores ≤6.9) (Fig. 1). One of these sites belonged to a public health organisation, while the other two were private websites. Within modalities, the highest compliance was for soft contact lenses (20% of the results; 2/10), followed by spectacles (10%; 1/10), with orthokeratology and atropine both at 0%.

There was no significant difference in the distribution of any of the readability scores across different modalities (FRES *p* = 0.76, FKGL *p* = 0.96, GFI *p* = 0.60, SMOG *p* = 0.64, CLI *p* = 0.99). Also, no significant correlations were found between the search rank position and any of the myopia modality readability indices (FRES *p* = 0.32, FKGL *p* = 0.67, GFI *p* = 0.26, SMOG *p* = 0.37, CLI *p* = 0.98).

### Myopia Management Products

The median scores for the 80 product website queries were: FRES 48.30 (IQR: 15.4, range 14.7–66.5); FKGL 9.9 (IQR: 3.0, range 6.3–14.3); GFI 11.4 (IQR: 3.5, range 5.6–16.1); SMOG 12.2 (IQR: 2.9, range 8.3–16.2) and CLI 12.2 (IQR: 2.6, range 8.7–16.2) (Table 2).

**Table 2:** Readability scores of patient education materials related to myopia products.

	Median readability scores (IQR) [range]						
Product	Word count	Words/Sentence	FRES	FKGL	GFI	SMOG	CLI
MiYOSMART® (spectacles)	878 (763)	11.2 (6.2)	42.7 (6.0)	10.1 (2.7)	11.2 (2.8)	12.8 (3.3)	12.2 (2.1)
	[954–1888]	[11.5–17.0]	[36.3–56.8]	[7.4–12.4]	[8.9–14.3]	[9.9–15.0]	[10.6–14.5]
Stellest® (spectacles)	852 (575)	12.1 (7.3)	52.6 (8.6)	8.8 (2.1)	10.7 (3.2)	11.7 (2.5)	11.9 (2.2)
	[172–2304]	[4.7–19.6]	[30.7–58.3]	[7.0–13.8]	[5.6–16.1]	[8.3–16.1]	[9.8–15.5]
MyoCare® (spectacles)	791 (335)	13.3 (3.4)	49.0 (5.0)	9.5 (1.5)	11.4 (2.5)	12.3 (1.8)	12.2 (0.9)
	[322–1631]	[9.2–17.3]	[35.2–55.1]	[8.3–12.6]	[9.8–15.5]	[11.2–14.9]	[11.4–14.8]
MiSight® 1 day (soft contact lenses)	642 (353)	14.2 (6.9)	50.1 (14.2)	9.9 (3.9)	11.4 (4.2)	12.2 (3.2)	12.5 (2.5)
	[341–3550]	[9.4–19.5]	[14.7–66.5]	[6.3–12.8]	[7.8–14.8]	[9.2–14.5]	[9.7–14.5]
ACUVUE® Abiliti® 1-Day (soft contact lenses)	392 (304)	13.2 (7.8)	43.4 (20.1)	11.0 (4.4)	12.6 (5.9)	13.1 (3.9)	12.5 (3.7)
	[230–4922]	[7.7–22.8]	[33.3–61.4]	[6.6–14.3]	[7.4–15.6]	[9.3–16.2]	[9.7–15.0]
NaturalVue® Multifocal 1 Day (soft contact lenses)	464 (227)	13.3 (3.8)	40.6 (13.9)	10.9 (0.3)	11.4 (2.1)	13.1 (1.4)	13.6 (2.6)
	[291–672]	[6.6–17.2]	[27.8–56.4]	[7.0–12.4]	[8.2–14.7]	[9.8–14.4]	[9.9–16.0]
ACUVUE® Abiliti® Overnight (orthokeratology)	592 (948)	8.5 (1.6)	54.8 (16.3)	7.6 (3.4)	8.4 (2.4)	10.6 (2.3)	10.3 (3.9)
	[227–16057]	[4.9–22.0]	[30.5–61.8]	[6.5–14.0]	[6.6–14.0]	[8.8–16.1]	[8.7–16.2]
Eikance (atropine)	1849 (1086)	11.3 (6.3)	48.5 (9.6)	9.5 (2.2)	12.5 (2.5)	12.1 (2.5)	12.4 (1.0)
	[396–3385]	[6.9–20.3]	[37.0–65.8]	[6.3–12.3]	[8.7–15.6]	[9.7–14.9]	[9.2–12.8]
*All products*	671 (786)	12.1 (6.7)	48.3 (15.4)	9.9 (3.0)	11.4 (3.5)	12.2 (2.9)	12.2 (2.6)
	[172–16057]	[4.7–22.8]	[14.7–66.5]	[6.3–14.3]	[5.6–16.1]	[8.3–16.2]	[8.7–16.2]

*FKGL* Flesch-Kincaid Grade Level, *FRES* Flesch Reading Ease Score, *GFI* Gunning Fog Index, *SMOG* Simple Measure of Gobbledygook Index, *CLI* Coleman Liau Index, *IQR* Interquartile Range.

**Fig. 2:**
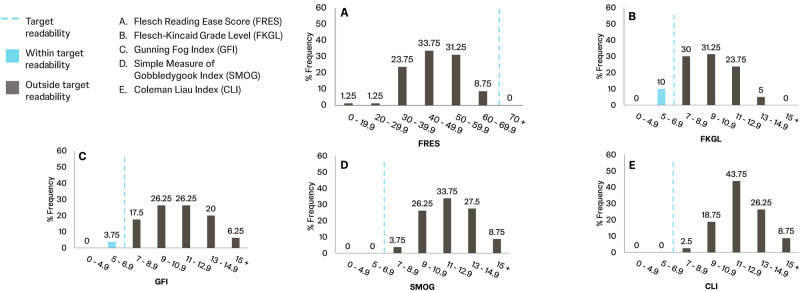
Percent frequency distribution of readability scores of online patient education materials for approved myopia management products. Blue bars indicate meets the target sixth-grade readability level; grey bars indicate does not meet sixth-grade readability.

Of the myopia product-related search results, 12.5% (10/80) of the websites complied with the recommended sixth-grade reading level. Of these 10 sites that were found to be compliant, eight met FKGL score targets and three sites met GFI score targets. There was one site that met the target readability of multiple scores (FKGL and GFI) and none met FRES, SMOG or CLI score targets (Fig. 2).

There was no significant difference in the distribution of any of the readability scores across different products. Only a weak positive correlation was found between search rank position and the SMOG (*r* = 0.27, *p* = 0.02) and GFI (*r* = 0.26, *p* = 0.02) indices, indicating that the top search rank positions were associated with slightly easier readability.

### *JAMA* Benchmarks

Of all the 120 search results, seven (5.83%) met the requirements to satisfy all four *JAMA* benchmark criteria, and all of these were from the AAO website (aao.org). Treatment modalities tended to have a greater proportion of patient education materials aligning with the *JAMA* criteria (85.7%) versus the product-oriented patient education materials (14.3%). Following this, three search results satisfied the requirements of three benchmarks, of which two (67%) were related to modality searches. The remaining 110 search results achieved two *JAMA* benchmarks or less (Table 3). The majority of websites (52.5%) met one *JAMA* benchmark, with authorship being the most fulfilled criterion (59.17%), followed by disclosure (17.5%), attribution (12.5%) and currency (9.17%). No significant correlations were found between *JAMA* benchmarks and readability for any of the search items across general myopia management enquiry, treatment modality or product query. An exception was a weak negative correlation between *JAMA* benchmarks and CLI score for both modality (*r* = −0.32, *p* = 0.045) and product (*r* = −0.22, *p* = 0.049) results, indicating that meeting more *JAMA* benchmarks is associated with slightly easier readability.

**Table 3:** Myopia management websites scored on the *Journal of the American Medical Association* (*JAMA*) benchmark criteria.

*JAMA* Benchmarks	Modalities		Products	
**Number of Benchmarks**	***N*** **=** **40**		***N*** **=** **80**	
	**Count**	**%**	**Count**	**%**
4	6	15	1	1.25
3	2	5	1	6.25
2	3	7.5	6	7.5
1	20	50	43	53.75
0	9	22.5	29	36.25
**Benchmark**	**Total**	**%**	**Total**	**%**
Attribution	9	22.5	6	7.5
Authorship	29	72.5	42	52.5
Currency	9	22.5	2	2.5
Disclosure	9	22.5	12	15

## Discussion

This study found that the majority of freely available online patient education materials related to myopia management modality and product queries were written above a US sixth-grade (6 years of formal schooling, corresponding to 11–12 years of age) reading level and do not meet all *JAMA* benchmarks. This difficulty in readability is consistent with previous studies that have assessed readability for various ocular treatments [30–33] as well as patient education materials in other health areas such as orthopaedics [34], geriatrics [35], sports medicine [36], sex education [37], cancer [38, 39] and COVID-19 [40, 41].

Of the 120 search results, none complied with the recommended sixth-grade reading level across all readability indices. Of all myopia modality-related search results, 7.5% (3/40) were found to be compliant with the FKGL score, with no sites meeting the FRES, GFI, SMOG or CLI score targets. Of the myopia product-related search results, 12.5% (10/80 sites) of the total websites complied with the recommended sixth-grade reading level. Of these, eight met FKGL score targets and three sites met GFI score targets, with one instance of a site meeting multiple readability targets (i.e., the sixth-grade reading level recommendation made by the USDHHS, corresponding with a FRES score ≥80 and FKGL, SMOG, GFI and CLI scores ≤6.9).

While a sixth-grade reading level is recommended by the USDHHS as well as other organisations such as the American Medical Association and National Institutes of Health in the USA, the average adult reads at an eighth-grade level (8 years of formal schooling, corresponding to 13–14 years of age) [34, 38, 42]. Extending target readability to an eighth-grade level corresponds to a FRES score ≥ 60, and FKGL, SMOG, GFI and CLI scores ≤8.9.

For modalities, broadening inclusion to an eighth-grade readability level and below resulted in compliance of 40%, 30%, 30% and 30% for spectacles, orthokeratology, atropine and soft contact lenses, respectively. Overall, compliance was 32.5% (13/40 sites) at the eighth-grade reading level versus 7.5% (3/40 sites) at the sixth-grade level. Similarly, for products, broadening inclusion up to an eight-grade reading level increased overall compliance to 40% (32/80 sites) from 12.5% (10/80 sites) at the sixth-grade level or below. These results show that there is a lack of accessible online patient education materials on myopia management treatments available at the recommended sixth-grade reading level.

The majority of websites did not meet all *JAMA* benchmarks of attribution, authorship, currency and disclosure. A total of seven websites, all from the AAO, satisfied all four benchmarks, while three websites met three benchmarks and the remainder achieved two or less. There was a weak positive relationship between product search rank and readability (SMOG *p* = 0.02, GFI *p* = 0.02) and a weak negative relationship between *JAMA* criteria of website searches and readability for both modality (CLI *p* = 0.045) and product (CLI *p* = 0.049).

A previous study that assessed the readability, accessibility and quality of online material for ‘myopia’ and ‘myopic degeneration’ using the internationally recognised DISCERN (instrument for quality of written patient health information) and the Health on the Net (HONcode) criteria tools, found that academic websites were significantly better quality compared with private websites [43]. The mean concise reading grade level was similar between both private and academic websites, and 7 out of 15 offered features catering to the visually impaired. One limitation is that this study only looked at 15 websites. Ravenstijn et al. assessed the perspectives of both patients and ophthalmologists regarding high myopia education, revealing that myopic patients rated the quality of information provided by the internet as higher than that provided in the clinic [44]. This may partly reflect time constraints in practice when discussing and educating patients in-office, as well as practitioner-centred communication styles [45].

When comparing these findings with other chronic ocular conditions such as glaucoma [46], uveitis [31] and dry eye [47], all appear to show a consistent lack of readability and quality across freely available online patient education materials for this range of conditions. These findings suggest that a significant proportion of patients are unlikely to find appropriate reading materials to improve their health literacy regarding their chronic ocular conditions, related medications and treatments. This has potentially important implications for patient care because such materials may modulate patient behaviour. For instance, with contact lens modalities such as orthokeratology and soft contact lenses, targeted patient information has been shown to improve lens care compliance and reduce the overall risk of red eye associated with lens usage [48].

Conversely, practitioners may find it challenging to address the information patients obtain online, and may struggle to decline requests for clinically inappropriate interventions based on such information [49]. Eye care providers can promote patient-centred care by reviewing credible online resources with their patients, as discussing online educational materials together may strengthen the patient-practitioner relationship [50].

Limitations of this study relate to the assumptions made by the readability formulas. Syllables per word and sentence length are major factors that determine readability across these formulas, but syntax and other contributing factors are ignored. Additionally, the use of medical or technical terminology is unavoidable to some degree, but the formulas used in this study do not account for this. The availability of software for finding plain language alternatives to technical terms could offer a feasible approach to communicate myopia management concepts, while aligning with recommended reading levels [51, 52].

Outside of the text itself, design elements such as layout, headings, bullet points and visuals also affect comprehension and health literacy, which were not assessed in this readability analysis. The Clear Communication Index (CCI) from the US Centers for Disease Control and Prevention incorporates quantitative and qualitative criteria to assess the clarity of patient education materials [53, 54]. While CCI scoring is less repeatable than objective measures such as FRES, FKGL, GFI, SMOG and CLI, future analysis of myopia management patient education materials could assess not only the readability, but also the clarity and communication quality of information.

While readability is a key component of health literacy, the present investigation did not account for cultural and linguistic competencies that may also contribute [55]. Additionally, search rankings were based on assumptions regarding what patients may search for and how. For instance, while materials designed for practitioners were screened out, patients will likely reach these websites, and could be frustrated by the assumed level of knowledge and subject complexity on these portals. Another factor to consider is the growing popularity of videos embedded into webpages as a source of education, which were not assessed here. Further, the current study also did not assess how websites addressed access for visually-impaired individuals.

While health information often has a higher readability level than is recommended, research shows that easier readability benefits outcomes for healthcare consumers. In a randomised controlled trial, a group receiving health information at a lower reading grade level had significantly higher recognition memory and ability to recognise previous information accurately than a control group [56]. Access to online health information after a diagnosis is useful to help healthcare consumers understand the services and treatments that are available. Even before a health consultation, two-thirds of patients have been found to search the internet [57] and there are case reports of using Google (google.com), also known as ‘Doctor Google’, as a diagnostic aid, particularly in cases of rare diseases [57–59]. Overall, access to online health information and easier readability helps empower patients.

It should also be acknowledged that patients are increasingly turning to social media for education [60]. Several studies have assessed the quality of myopia control education on such platforms. For example, a study that assessed the content of the top TikTok (tiktok.com) videos in China on myopia using the DISCERN criteria found that the content had moderate to poor reliability across video sources, with many documented instances of misinformation [61]. Likewise, Boz and Kocamis analysed the quality of the first 100 YouTube (youtube.com) videos in response to a ‘myopia control’ prompt [62], and also found a paucity of quality output, with very little information on lifestyle modifications [62]. In contrast to these findings, a more targeted family health education weekly messaging programme on WeChat (wechat.com) resulted in a slight decrease in myopia incidence over 2 years [63].

In conclusion, the majority of freely available online patient education materials relating to myopia management modalities and products are written above the recommended sixth-grade reading level and do not meet all of the *JAMA* benchmarks. Websites that appear as the top search results do not necessarily have easier readability. The readability of online patient education materials should be a key consideration, as it may influence access to treatment and patient outcomes. This presents an opportunity to educate and advance patient awareness on the need to manage myopia.

## Data Availability

The URLs of all collected search results are available in Appendix Tables 1 and 2.

## Appendix Table 1. Modality websites used in analysis, ordered by search rank appearance from 1 to 10 within each modality

**Table:**

Modality	URL
Orthokeratology	https://www.mykidsvision.org/knowledge-centre/ortho-k-for-myopia-control
Orthokeratology	https://aaomc.org/patients/what-is-myopia/
Orthokeratology	https://www.aao.org/eye-health/diseases/myopia-control-in-children
Orthokeratology	https://www.orthoktoronto.com/what-is-myopia-management?_sm_nck=1
Orthokeratology	https://www.ocuco.com/resources/blogs/orthokeratology-for-myopia-control
Orthokeratology	https://www.mykidsvision.org/knowledge-centre/orthok-for-myopia-control-in-children
Orthokeratology	https://minamiaoyama.or.jp/official/wp-content/uploads/ortho_en.pdf
Orthokeratology	https://www.accessdata.fda.gov/cdrh_docs/pdf/p010062d.pdf
Orthokeratology	https://pi.bausch.com/globalassets/pdf/packageinserts/vision-care/bostonlensmaterials/pib-and-pi/Patient-Information-Booklet-Potential.pdf
Orthokeratology	https://drbleonard.com/eye-diseases/myopia/what-is-orthokeratology-treatment/
Spectacles	https://www.mykidsvision.org/knowledge-centre/all-about-eye-glasses-for-myopia-control
Spectacles	https://www.aao.org/eye-health/diseases/myopia-control-in-children
Spectacles	https://www.mykidsvision.org/knowledge-centre/eye-glasses-for-myopia-control-in-teenagers
Spectacles	https://www.mykidsvision.org/knowledge-centre/which-is-the-best-option-for-myopia-control
Spectacles	https://www.hoyavision.com/au/for-spectacle-wearers/miyosmart/
Spectacles	https://www.zeiss.com/vision-care/en/need-new-lenses/myopia-management-lenses.html
Spectacles	https://www.opsm.com.au/myopia-control-lenses
Spectacles	https://bullseyeclinic.com.au/myopia-management/
Spectacles	https://davidshanahan.com.au/myopia-control-treatment-that-works/
Spectacles	https://www.canterburyeyecare.com.au/myopiamanagement/
Atropine	https://www.mykidsvision.org/knowledge-centre/atropine-eye-drops-for-myopia-control-in-children
Atropine	https://www.aao.org/eyenet/article/how-to-use-low-dose-atropine-to-slow-myopic-progre
Atropine	https://www.aao.org/eye-health/diseases/myopia-control-in-children
Atropine	https://www.ocula.co.nz/my-treatment/atropine-for-myopia-control/
Atropine	https://www.mykidsvision.org/knowledge-centre/which-is-the-best-option-for-myopia-control
Atropine	https://www.aao.org/eye-health/news/low-dose-atropine-kids-with-myopia
Atropine	https://specialtyeye.com/how-do-atropine-eye-drops-help-control-myopia-in-kids/
Atropine	https://drbishop.com/how-does-atropine-for-myopia-control-work/
Atropine	https://www.mykidsvision.org/knowledge-centre/atropine-eye-drops-for-myopia-in-young-adults
Atropine	https://www.eyefocus.com.au/patient-information
Soft contact lenses	https://www.mykidsvision.org/knowledge-centre/how-do-myopia-control-soft-contact-lenses-work
Soft contact lenses	https://www.mykidsvision.org/knowledge-centre/soft-contact-lenses-for-myopia-control-in-children
Soft contact lenses	https://misight.com/
Soft contact lenses	https://coopervision.com/myopia-management
Soft contact lenses	https://www.aao.org/eye-health/tips-prevention/misight-orthok-atropine-myopia-nearsighted-child
Soft contact lenses	https://www.mykidsvision.org/knowledge-centre/soft-contact-lenses-for-myopia-control-in-young-adults#:~:text=Soft%20myopia%20control%20contact%20lenses,of%2Dfocus%20and%20multifocal%20designs.
Soft contact lenses	https://www.optometrists.org/childrens-vision/guide-to-pediatric-eye-conditions/what-is-myopia-management/myopia-management-which-contact-lenses/
Soft contact lenses	https://royaldevon.nhs.uk/media/g5vondnv/patient-information-leaflet-myopia-and-myopia-management-rde-22-045-001.pdf
Soft contact lenses	https://www.kidseyecare.com/services/myopia-control/
Soft contact lenses	https://www.ancasterfamilyeyecare.com/misight-contact-lenses-for-myopia-management/

## Appendix Table 2. Product websites used in analysis, ordered by search rank appearance from 1 to 10 within each product

**Table:**

Product	URL
MiYOSMART®	https://www.hoyavision.com/en-ca/vision-products/miyosmart/
MiYOSMART®	https://www.hoyavision.com/en-ca/for-eyeglass-wearer/miyosmart/
MiYOSMART®	https://www.southvanoptometry.com/pdf/MiyoSmart-Information.pdf
MiYOSMART®	https://info.hoyavision.com/hubfs/HOYA%20Canada%202023/(MiYOSMART%20ECP%20Brochure%20-%20EN)%20HOYA%20MiYOSMART%20Complete%20Portfolio%20ECP%20Brochure%20May%202023.pdf?hsCtaTracking=7544c305-2329-4241-971d-5729f1cd9e40%7C2692f687-331e-4fb5-aa71-d0884cbab3e9
MiYOSMART®	https://www.moussaeyedoctor.com/storage/app/media/miyosmart-ecp-brochure-final-eng-single-page-email-v3.pdf
MiYOSMART®	https://www.clarityoptometry.ca/eye-care-services/myopia-management-optometrist/miyosmart-for-myopia-management/
MiYOSMART®	https://www.mississaugavisioncentre.com/eye-care-services/myopia-management-optometrist/what-treatments-are-used-in-myopia-management/miyosmart-for-myopia-management/miyosmart-lenses-for-myopia-control/
MiYOSMART®	https://heliooptometry.ca/miyosmart-myopia-treatment-eyeglasses-for-kids
MiYOSMART®	https://www.360eyecare.ca/miyosmart/
MiYOSMART®	https://eye-bar.ca/miyosmart-eyeglass-lenses-for-myopia-control-treatment-in-kids
Stellest®	https://www.mykidsvision.org/knowledge-centre/essilor-stellest-glasses-lenses-parents-guide
Stellest®	https://www.essilor.com/ca-en/products/stellest/
Stellest®	https://heliooptometry.ca/blog/who-can-benefit-the-most-from-stellest-myopia-control-eyeglass-lenses
Stellest®	https://d15k2d11r6t6rl.cloudfront.net/public/users/Integrators/BeeProAgency/20036_8016/2021%20Stellest_Brochure_A4%20Short%20Version_New%20%28final%2020211103%29.pdf
Stellest®	https://www.yourvision.ca/myopia-control/
Stellest®	https://drbishop.com/service/myopia-management/
Stellest®	https://ecp.essilor-pro.com/gb/essilor-lenses/myopia-management
Stellest®	https://www.visionexpress.com/brands/stellest-lenses
Stellest®	https://www.essilor.ca/content/dam/essilor-ca/f3/Stellest%20Brochure_BtoB_2023%20EN%20LR.pdf
Stellest®	https://betasimplicity.com/blog_stellest_techreview.html
MyoCare®	https://www.zeiss.com/vision-care/en/need-new-lenses/myopia-management-lenses.html
MyoCare®	https://www.zeiss.ca/vision-care/en/eye-care-professionals/lenses/lenses-for-every-need/lenses-to-manage-the-progression-of-myopia-in-children.html
MyoCare®	https://centraloptometry.com/myopia-control-management-treatment/
MyoCare®	https://abbeyeyecare.ca/zeiss-myocare-myopia-management-lenses/
MyoCare®	https://www.woptics.sg/happenings/article/zeiss-myocare-myopia-control-lenses
MyoCare®	https://www.avenueeyecare.com/new-myocare-myocare-s-myopia-management-spectacle-lenses-available/
MyoCare®	https://evershineoptical.com.sg/2023/03/20/zeiss-myocare-new-myopia-control-lens/
MyoCare®	https://matadoreyeworks.com/eyeglasssunglasstechnology/myopia-management/
MyoCare®	https://myeyecare.ca/service/myopia-control/
MyoCare®	https://calgaryfamilyeyedoctors.com/what-is-myopia-control/
MiSight® 1 day	https://coopervision.com/contact-lenses/misight-1-day
MiSight® 1 day	https://misight.com/frequently-asked-questions
MiSight® 1 day	https://coopervision.com/myopia-management
MiSight® 1 day	https://misight.com/
MiSight® 1 day	https://coopervision.ca/myopia-management
MiSight® 1 day	https://www.kidseyecare.com/services/myopia-control/
MiSight® 1 day	https://eyefreedom.com/misight-1-day-contact-lenses-myopia-treatment/
MiSight® 1 day	https://www.miamicontactlens.com/misight-lenses/
MiSight® 1 day	https://www.eyelink.com/eye-care-services/myopia-management-optometrist/misight-contact-lenses-for-myopia-management/
MiSight® 1 day	https://all-eye-care.com/eye-care/misight-myopia-management-the-first-fda-endorsed-myopia-control-for-kids/
ACUVUE® Abiliti® 1-Day	https://www.seeyourabiliti.com/sg/parents/abiliti-portfolio/abiliti-1-day
ACUVUE® Abiliti® 1-Day	https://www.jjvision.com/press-release/johnson-johnson-vision-receives-approval-canada-acuvuer-abilititm-1-day-soft
ACUVUE® Abiliti® 1-Day	https://d2lxochaelxdve.cloudfront.net/2022-11/Abiliti%201-Day%20Instruction%20Guides.pdf
ACUVUE® Abiliti® 1-Day	https://www.clearvisionforyou.com/en-ca/myopia/abiliti-1-day/
ACUVUE® Abiliti® 1-Day	https://www.clearvisionforyou.com/en-ca/myopia/myopia-treatment/
ACUVUE® Abiliti® 1-Day	https://www.thompsonoptics.com/eye-care-services/myopia-management-optometrist/abiliti-1day-lenses-for-myopia-management/
ACUVUE® Abiliti® 1-Day	https://www.dolmaneyecare.com/myopia-management-optometrist/abiliti-1day-lenses-for-myopia-management/
ACUVUE® Abiliti® 1-Day	https://www.eyetrusteyecare.ca/introducing-acuvue-abiliti-contact-lenses/
ACUVUE® Abiliti® 1-Day	https://www.180optometry.com/abiliti-1-day-soft-disposable-contacts
ACUVUE® Abiliti® 1-Day	https://www.lifetime-eyecare.ca/eye-care-services/myopia-management-optometrist/ortho-k-the-safe-way-to-sleep-in-contact-lenses/abiliti-1day-lenses-for-myopia-management/
NaturalVue® Multifocal 1 Day	https://vtivision.com/practitioner/products/enhanced-multifocal/
NaturalVue® Multifocal 1 Day	https://www.visualsymptomstreatmentcenter.com/eye-care-services/myopia-management-optometrist/naturalvue-contact-lenses-for-myopia-management/
NaturalVue® Multifocal 1 Day	https://www.thespectrumeyecentre.com/technology/naturalvue.php
NaturalVue® Multifocal 1 Day	https://vtivision.com/patient/myopia/
NaturalVue® Multifocal 1 Day	https://drbleonard.com/eye-wear/contact-lenses/naturalvue-contact-lenses/
NaturalVue® Multifocal 1 Day	https://www.eyeworksgroup.com/eye-care-services/orthokeratology-crt-vst/myopia-management-optometrist/naturalvue-multifocal-lenses-for-myopia-control/
NaturalVue® Multifocal 1 Day	https://www.peterivins.co.uk/myopia-management-clinic/naturalvue/
NaturalVue® Multifocal 1 Day	https://www.jamestracey.com/eye-care-services/myopia-management-optometrist/naturalvue-multifocal-lenses-for-myopia-control/
NaturalVue® Multifocal 1 Day	https://www.aspirevisioncare.com/naturalvue-multifocal-lenses-for-myopia-control/
NaturalVue® Multifocal 1 Day	https://eatonopt.com/vision-care-products/myopia-management/
ACUVUE® Abiliti® Overnight	https://www.seeyourabiliti.com/sg/parents/abiliti-portfolio/abiliti-overnight
ACUVUE® Abiliti® Overnight	https://www.clearvisionforyou.com/en-ca/myopia/abiliti-overnight/
ACUVUE® Abiliti® Overnight	https://www.clearvisionforyou.com/en-ca/myopia/myopia-treatment/
ACUVUE® Abiliti® Overnight	https://www.jjvision.com/press-release/johnson-johnson-vision-receives-approval-canada-acuvuer-abilititm-overnight
ACUVUE® Abiliti® Overnight	https://d2lxochaelxdve.cloudfront.net/2022-11/Abiliti%20Overnight%20Instruction%20Guides.pdf
ACUVUE® Abiliti® Overnight	https://www.clearvisionforyou.com/en-us/myopia/myopia-management/#
ACUVUE® Abiliti® Overnight	https://d2lxochaelxdve.cloudfront.net/2023-10/AbilitiOvernightIFUsUS-new.pdf
ACUVUE® Abiliti® Overnight	https://www.richmondhilleye.com/eye-care-services/myopia-control-for-children/abiliti-lenses-for-myopia-treatment/
ACUVUE® Abiliti® Overnight	https://www.nyeyedocs.com/storage/app/media/abiliti-brochure-us-approved-pp2022abl4071-1.pdf
ACUVUE® Abiliti® Overnight	https://www.orthoklove.com/wp-content/uploads/2024/03/Abiliti-Overnight-Patient-FAQ-Handout-APPROVED-PP2023ABL4226-copy.pdf
Eikance	https://www.medsafe.govt.nz/profs/datasheet/e/EikanceEyeDrops.pdf
Eikance	https://www.nps.org.au/medicine-finder/eikance-0-01
Eikance	https://www.healthdirect.gov.au/medicines/brand/amt,1574941000168107/eikance
Eikance	https://www.mykidsvision.org/knowledge-centre/atropine-eye-drops-for-myopia-control-in-children
Eikance	https://www.aao.org/eyenet/article/how-to-use-low-dose-atropine-to-slow-myopic-progre
Eikance	https://mydr.com.au/medicines/eikance-0-01-eye-drops/
Eikance	https://www.ocula.co.nz/my-treatment/atropine-for-myopia-control/
Eikance	https://collinandkirk.com.au/myopia-and-myopia-control/
Eikance	https://www.eyecareconcepts.com.au/blog/where-to-buy-001-atropine-eye-drops
Eikance	https://ranzco.edu/wp-content/uploads/2022/05/RANZCO-Position-Statement-Progressive-Myopia-in-Childhood_2022.pdf
